# 体外膜肺氧合在复杂气管支气管手术中的应用：系列病例报道和文献综述

**DOI:** 10.3779/j.issn.1009-3419.2024.101.22

**Published:** 2024-09-20

**Authors:** Chen SHU, Peilong BAO, Yunfeng NI, Jie LEI, Xiaolong YAN, Nianlin XIE, Jinbo ZHAO

**Affiliations:** 710038 西安，空军军医大学唐都医院胸腔外科; Department of Thoracic Surgery, Tangdu Hospital, Air Force Medical University, Xi'an 710038, China

**Keywords:** 体外膜肺氧合技术, 气管支气管, 气道手术, Extracorporeal membrane pulmonary oxygenation, Tracheobronchial, Airway surgery

## Abstract

复杂气管支气管手术（tracheobronchial surgery, TBS）的气道管理仍然是胸外科手术中的难点。除肺移植手术之外，体外膜肺氧合技术（extracorporeal membrane pulmonary oxygenation, ECMO）在胸外科手术中应用较少。为了探究ECMO在复杂TBS中的安全性和有效性，本研究收集了2019年5月至2024年6月空军军医大学唐都医院胸腔外科在ECMO支持下的复杂气管、支气管重建手术患者共5例，其中气管肿瘤4例（长段气管切除重建或隆突切除重建），气管断裂导致气道急性梗阻1例。5例患者全部采用静脉到静脉ECMO（veno-venous ECMO, V-V ECMO）模式，2例患者采用了全身肝素化，3例患者未进行全身肝素化，仅通过ECMO肝素涂层管路维持。术后4例患者恢复良好，1例患者因免疫相关性肺炎于术后1个月死亡。对于复杂TBS（长段气管切除、气管断裂导致气道急性梗阻或隆突切除重建），或在紧急状况下（气管狭窄，存在窒息风险），ECMO能够提供较好的支持和保障。

## Abstract (en)

Airway management in complex tracheobronchial surgery (TBS) remains a challenge in thoracic surgery. The use of extracorporeal membrane pulmonary oxygenation (ECMO) in thoracic surgery is rather rare, except for lung transplantation. To report the safety and efficacy of ECMO in complex TBS, a total of 5 patients with tracheobronchial and bronchial reconstructive surgery supported by ECMO in the Department of Thoracic Surgery of Tangdu Hospital, Air Force Medical University from May 2019 to June 2024 were collected. Among them, 4 cases of tracheal tumor (including long-segment trachea resection and reconstruction, or carinal resection and reconstruction) and 1 case of acute airway obstruction caused by tracheal rupture were included, all of which were performed in veno-venous ECMO (V-V ECMO) mode. Systemic heparinization was used in 2 patients, and anticoagulation was not performed in 3 patients, which were maintained only by ECMO heparin-coated lines. 4 patients recovered well after surgery, and 1 patient died 1 month after surgery due to immune-related pneumonia. For complex TBS, or in emergency situations (tracheal stenosis with risk of asphyxiation), ECMO can provide adequate support and safeguard.

气管支气管手术（tracheobronchial surgery, TBS）对麻醉策略提出显著挑战。由于气管手术中手术部位和麻醉共用气道，因此，常规气管插管麻醉对于术者要求较高，要在保持患者充足的氧合和通气的同时，完成气管的切除重建。非插管麻醉包括：自主呼吸手术，体外膜肺氧合（extracorporeal membrane oxygenation, ECMO）支持下手术，有利于术者的操作和维持清晰的术野，可能在气管支气管切除重建中具有一定的优势。非插管自主呼吸支持下的麻醉方式被证明在颈段气管以及有选择的胸段气管切除重建中安全有效，通过先期气管镜下切除大部分病变后，保留自主呼吸，在颈部或胸腔镜下可安全完成气管切除重建。但是，对于长段气管切除或者复杂气管切除重建仍存在着一定的风险^[1,2]^。ECMO是一种体外生命支持技术，由于其能提供安全充足的氧合、维持良好的手术野、便于麻醉管理和手术操作等优势，因而在TBS中得到越来越广泛的应用^[3,4]^。本研究旨在回顾空军军医大学唐都医院胸腔外科在ECMO支持下完成复杂TBS的治疗经验。

## 1 资料与方法

回顾性选择空军军医大学唐都医院胸腔外科2019年5月至2024年6月在ECMO支持下完成复杂TBS的患者共5例。所有患者均在ECMO支持下接受了TBS治疗。所有气管肿瘤和气管外伤的病例均结合影像学及病理学的结果进行诊断。其中4例气管肿瘤患者通过支气管镜及组织病理学结果进行确诊，1例气管外伤患者通过胸部计算机断层扫描（computed tomography, CT）联合三维重建及支气管镜进行确诊。本研究中所有患者均接受了评估，包括完整的病史资料、体格检查、血常规、生化、胸部X线片、胸部CT、纤维支气管镜、病理学检查等。

经空军军医大学唐都医院伦理委员会批准（批准号：No.202112-05），并获得患者及家属的知情同意，本研究回顾了这5例患者的临床特点及总结ECMO在TBS中的应用经验。

## 2 结果

在本研究中，5例ECMO支持下完成复杂TBS的患者临床资料分别见表1和表2。所有患者中包含男性2例，女性3例，年龄34-62（46.0±12.7）岁。5例患者中有2例患者有吸烟史，其中1例有20年的吸烟史；另1例为偶尔吸烟者。所有患者的病变部位分别为气管下段（n=3）（其中1例患者病变累及膜部，1例患者病变累及右主支气管）、气管中段（n=1）、隆突（n=1），均无位于气管上段。本组5例患者中有3例病变类型最后确诊为气管腺样囊性癌（tracheal adenoid cystic carcinoma, TACC），包含1例复发性腺样囊性癌，上述3例患者均在术前接受经支气管镜下减瘤手术，其中复发性腺样囊性癌患者接受了术后化疗（4个周期，培美曲塞+奈达铂）。另1例气管肿瘤患者最后诊断为隆突大细胞肺神经内分泌癌，该患者术前接受了4个周期的新辅助化疗联合免疫治疗（依托泊苷+卡铂+斯鲁利单抗）。1例患者最后诊断为气管断裂导致气道急性梗阻，术前在支气管镜下行球囊扩张术，效果欠佳。3例患者（长段气管切除重建、气管断裂致气道急性梗阻及隆突切除重建）的术前胸部CT及支气管镜图像见图1。所有患者均无高血压、糖尿病等合并症。

**表1:** 患者的临床资料

Patient No.	Gender	Age (yr)	Smoking status	Location	Disease type	Treatment	Complications
1	Female	35	No	LT+TM	TACC	BTR	No
2	Male	34	Yes	MT	AAO	BBD	No
3	Female	38	No	LT+RMB	TACC	BTR	No
4	Female	61	No	LT	RTACC	BTR+CT	No
5	Male	62	Yes	TC	LCNC	CT+Sluli	No

LT: lower trachea; TM: tracheal membranes; MT: middle trachea; RMB: right main bronchus; TC: tracheal carina; TACC: tracheal adenoid cystic carcinoma; AAO: acute airway obstruction; RTACC: recurrent tracheal adenoid cystic carcinoma; LCNC: large cell neuroendocrine carcinoma; BTR: bronchoscopic tumor resection; BBD: bronchoscopic balloon dilation; CT: chemotherapy; Sluli: Slulimumab.

**表2:** 患者行ECMO支持的围术期管理和转归

Patient No.	Incisions	Surgical procedure	ECMO mode	ECMO support time (min)	Postoperative complication	Follow up (mon)	Outcome
1	RPLT	TREEA	VV-ECMO	60	No	32	Alive with PM
2	MS	TREEA	VV-ECMO	60	No	31	Alive
3	RPLT	TR+RMBT+SLT+CRR	VV-ECMO	90	Bleeding	33	Alive with PM
4	RPLT	TR+SLT+CRR	VV-ECMO	80	No	29	Alive
5	RPLT	CRR	VV-ECMO	100	IRP	2	Died

RPLT: right posterior lateral thoracotomy; MS: median sternotomy; TR: tracheal resection; TREEA: tracheal resection and end-to-end anastomosis; RMBT: right main bronchus resection; SLT: sleeve lobectomy; CRR: carinal resection and reconstruction; ECMO: extracorporeal membrane oxygenation; VV-ECMO: veno-venous ECMO; IRP: immune-related pneumonia; PM: pulmonary metastasis.

**图1:**
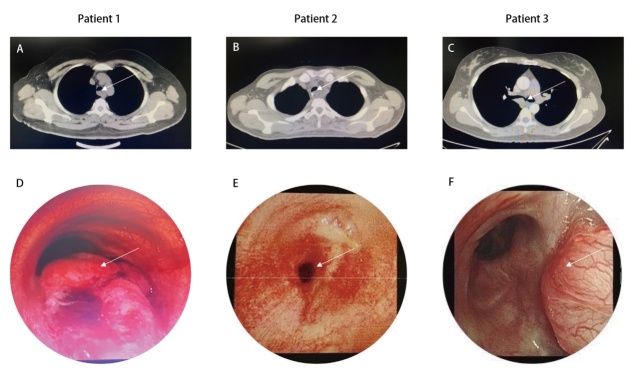
3例复杂TBS患者术前胸部CT、支气管镜图像。A、B、C：分别为长段气管切除重建、气道断裂导致急性气道梗阻、隆突切除重建患者术前胸部CT图像（白色箭头为病变部位）；D、E、F：分别为3例患者术前支气管镜图像（白色箭头为病变部位）。

在本研究中，所有患者均采用开胸手术，4例患者经右后外侧切口入路，1例患者因气管病变位置过深，颈部切口受限，故采用胸部正中切口。对于长段气管切除重建的患者，肿瘤累及隆突上气管及右主支气管总长度约6 cm，且因右主支气管切缘阳性，故最终行气管下段、右主支气管肿瘤切除，伴右肺上叶切除及隆突重建术。气管断裂导致气道急性梗阻患者术前出现2型呼吸衰竭后行急诊手术，术中证实为气道断裂，术中切除瘢痕气管后吻合气管残端。隆突切除重建患者在术中切除隆突范围为主气管、左右主支气管各1.5 cm，后将左主支气管与主气管2/3、右主支气管与主气管1/3分别吻合（手术示意图见图2）。所有患者术中ECMO支持均采用静脉到静脉ECMO（veno-venous ECMO, V-V ECMO）转流模式，即全身麻醉后，首先给予肝素（50 IU/kg），在超声引导下于右侧股静脉和右侧颈内静脉置管，建立VV-ECMO循环通路。ECMO流量稳定在3 L/min左右，转速约为3000 rpm，测定活化凝血时间（activated clotting time, ACT）≥136 s，术中通过ACT值动态调整肝素用量。围手术期的ACT、血气分析等监测结果见表3。手术过程中，2例患者采用了全身肝素化；3例患者未进行全身肝素化，未加用其他抗凝药物，仅通过ECMO肝素涂层管路维持。手术完成后，待患者血氧等指标稳定，在术中通气恢复，逐渐减少流量至1.0 L/min，监测动脉血气氧合指数>200后撤去ECMO，患者ECMO支持时间为60-100（78.0±17.9）min。所有患者均在术后恢复机械通气后撤去ECMO，带气管插管入重症监护室继续治疗。3例患者（长段气管切除重建、气管断裂导致气道急性梗阻及隆突切除重建）术后复查胸部CT及支气管镜图像见图3。术后所有患者未出现感染，血常规、降钙素原等检验结果见表3。1例患者考虑术前全身肝素化后凝血功能欠佳致术后胸腔内出血，经开胸探查止血后好转，1例患者因出现免疫相关性肺炎于术后1个月死亡，其余4例患者均存活出院，2年生存率为80%（4/5）。术后随访过程中2例患者出现肺转移瘤可能，所有患者随访至2024年6月10日，随访时间为2-33（25.4±11.7）个月。

**图2:**
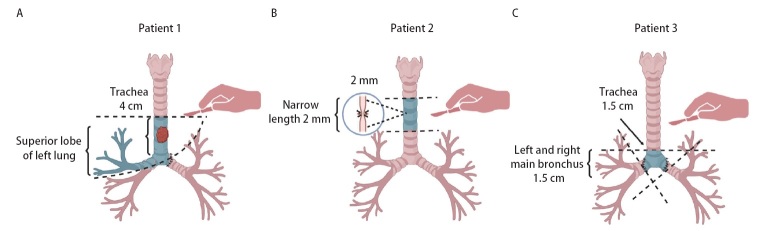
3例复杂TBS患者手术示意图。A、B、C：分别为长段气管切除重建、气道断裂导致急性气道梗阻、隆突切除重建患者手术示意图，其中浅蓝色部分为切除气管部分。

**图3:**
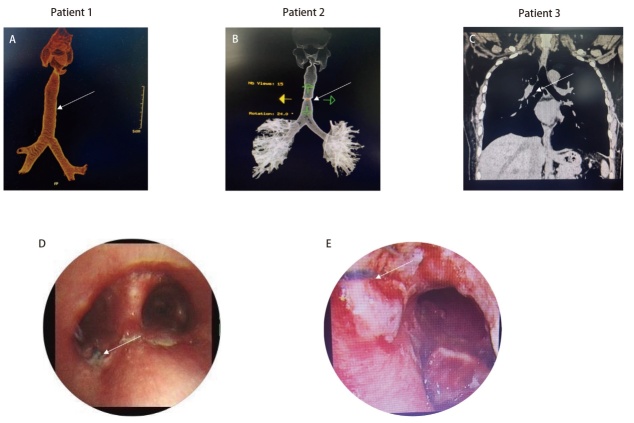
复杂TBS患者术后胸部CT及支气管镜图像。A：长段气管切除重建患者术后剩余气管长度；B：气道断裂导致急性气道梗阻患者术后气管通畅；C：隆突切除重建患者术后气管及双侧支气管通畅；D、E：长段气管切除重建、隆突切除重建患者术后支气管镜图像（白色箭头为手术缝线）。

**表3:** 患者术中术后的ECMO相关指标及主要检验结果

Patient No.	OI^a^ (mmHg)	ACT^b^ (s)	ABG^c^	BALF	PCT (ng/mL)	RBT
1	312	130-160	pH 7.43-7.55; pCO_2_ 25-41 mmHg; pO_2_ 75-270 mmHg; HCT 37%-40%; SaO_2_ 98%-100%; HCO_3_^-^ 21.4-27.2 mmol/L	Negative	0.06	WBC 5.33×10^9^/L; LYMPH 1.19×10^9^/L; RBC 3.44×10^9^/L; HGB 111 g/L
2	400	120-180	pH 7.35-7.58; pCO_2_ 31-61 mmHg; pO_2_ 96-415 mmHg; HCT 28%-35%; SaO_2_ 95%-99%; HCO_3_^-^ 29.1-33.7 mmol/L	NA	0.10	WBC 9.63×10^9^/L; LYMPH 0.36×10^9^/L; RBC 3.44×10^9^/L; HGB 111 g/L
3	258	130-170	pH 7.28-7.46; pCO_2_ 30-51 mmHg; pO_2_ 62-255 mmHg; HCT 19%-38%; SaO_2_ 92%-99%; HCO_3_^-^ 20.9-27.0 mmol/L	Negative	0.03	WBC 8.84×10^9^/L; LYMPH 0.39×10^9^/L; RBC 3.72×10^9^/L; HGB 115 g/L
4	375	120-190	pH 7.42-7.53; pCO_2_ 22-39 mmHg; pO_2_ 120-461 mmHg; HCT 23%-37%; SaO_2_ 90%-100%; HCO_3_^-^ 18.4-25.3 mmol/L	Negative	0.36	WBC 8.46×10^9^/L; LYMPH 0.47×10^9^/L; RBC 2.72×10^9^/L; HGB 89 g/L
5	387	140-170	pH 7.37-7.51; pCO_2_ 25-45 mmHg; pO_2_ 93-188 mmHg; HCT 22%-39%; SaO_2_ 95%-99%; HCO_3_^-^ 20.9-27.0 mmol/L	Negative	0.05	WBC 4.24×10^9^/L; LYMPH 0.31×10^9^/L; RBC 3.01×10^9^/L; HGB 94 g/L

^a^OI before ECMO was removed; ^b^Range of intraoperative ACT values; ^c^Results of intraoperative ABG. OI: oxygenation index; ACT: activated clotting time; ABG: arterial blood gas; HCT: hematocrit; BALF: bronchoalveolar lavage fluid; PCT: procalcitonin; RBT: routine blood test; WBC: white blood cell; LYMPH: lymphocyte; RBC: red blood cell; HGB: hemoglobin; NA: not available.

## 3 讨论

复杂TBS麻醉中的气道管理和充足的氧合通常需要多学科的讨论和个性化方案设计。传统的全身麻醉，术中进行台上插管维持氧合依然是经典的气管手术麻醉方法；对于术中切除病变范围较小且身体状况较好的患者，可以考虑采用保留自主呼吸非插管麻醉下的气管切除重建手术。但术中应当准备好气管插管、胸腔镜下插管以及机械通气设备，以便在术中出现血氧饱和度较低无法维持，持续的二氧化碳潴留、呼吸性酸中毒，或者突发无法控制出血时紧急进行气管插管来恢复机械通气^[1,2,5]^。对于复杂气管切除重建手术，或者患者处于窒息等高风险状态下，保留自主呼吸非插管仍存在着一定的风险。因此对于长段气管切除或者复杂气管切除重建，ECMO具有较好的优势。He等^[6]^报道了1例ECMO支持下的长段气管切除重建，撤去ECMO后血流动力学稳定，且存活出院。在Suzuki等^[7]^报道的34例ECMO支持下的气管支气管肿瘤中，25例患者存活出院（73.25%）。本研究回顾了空军军医大学唐都医院胸腔外科5例在VV-ECMO辅助下的复杂TBS患者的诊断及治疗经验，存活出院率为80%（4/5），与国内外研究报道相近。所有患者中ECMO支持下4例气管肿瘤被报道，1例患者为隆突肿瘤，经ECMO支持在术中撤去ECMO，术后无ECMO相关并发症，但在术后1个月因免疫相关性肺炎而死亡。其余3例患者最后诊断为TACC。TACC具有以下特征：首先，TACC是一种上皮来源的低度恶性的涎腺性肿瘤，通常无吸烟史^[8]^；其次，TACC初发时通常无特殊临床表现，在出现气道阻塞症状时可通过支气管镜及病理活检明确诊断；第三，由于TACC具有黏膜下生长的特性，手术后切缘阳性率及手术后复发率可达30%以上^[9,10]^。有研究^[11]^表明，TACC术后较易复发及转移，且肺是最常见的转移部位。上述3例TACC患者中包含1例长段气管切除，该患者气管下段及右主支气管病变长度超过6 cm，而研究^[12]^表明成人气管切除长度超过6 cm对实现气管端端吻合是一个严峻的挑战。该患者在ECMO支持下通过松解气管周围组织来实现无张力吻合，并且切缘阴性，但是在随访中发现该患者在术后3年出现肺转移瘤。另外2例TACC患者也出现复发性TACC及术后第3年发现肺部转移，经回顾文献，并无明确ECMO术后可引起远处转移的研究，因此我们认为转移不一定和ECMO使用存在相关性，需积累更多病例来进一步明确ECMO使用和气管肿瘤转移之间的关系，最后我们考虑这可能与TACC自身黏膜下生长及易复发等因素密切相关。因此我们建议患者无论是否出现呼吸道症状，均应定期复查胸部CT或支气管镜，做到早诊断早治疗。外伤导致的气管损伤甚至气管断裂可能引起气道急性梗阻，常伴有肺部挫伤、胸腔积血等并发症，可导致严重酸中毒、缺氧、高碳血症，出现严重的呼吸困难甚至循环功能障碍。当出现血流动力学不稳定或者机械通气效果不佳等紧急情况时，ECMO可以提供充足的氧合，有效保证手术的顺利进行，是一种可行的抢救治疗方法。Schmoekel等^[13]^使用ECMO救治了1例严重支气管损伤的患者，术后患者出现脓胸，经救治后好转出院。Clark等^[14]^报道了1例外伤致颈部气管近横断性损伤患者，经ECMO支持后好转出院，1年后复查气管通畅，无并发症发生。在本研究中，1例气管断裂导致气道急性梗阻的患者，因术前出现2型呼吸衰竭而在ECMO支持下紧急手术，术中撤去ECMO，恢复良好出院。

自1992至2022年，共有168例使用ECMO支持的TBS病例被文献报道，其中在2015至2022年期间，有135例患者接受了VV-ECMO支持，因此VV-ECMO被认为是最常见的转流模式^[15]^。常见的ECMO转流模式还包括静脉到动脉ECMO（veno-arterial ECMO, VA-ECMO）。VA-ECMO主要用于术前或者术中可能出现循环系统不稳定的情况，VA-ECMO能够部分提供循环支持。对于右心室功能正常的患者，VV-ECMO是首选，因为它避免了胸骨正中切开的需要，比体外循环引起的肝素化更少，并且动脉插管相关并发症（包括出血、缺血和栓塞）的风险更低^[16]^。一项多中心回顾性研究^[17]^报道了20例VV-ECMO和16例VA-ECMO患者，VV-ECMO的中位支持时间为78 min，这与我们的ECMO支持时间的数据一致。而VA-ECMO的中位支持时间为65 min，这36例患者中有2例发生插管相关并发症。有团队实施了1例VV-ECMO辅助下机器人气管切除重建，患者于术后10 d出院，且无并发症发生，出院后复查支气管镜提示气管吻合正常^[18]^。本研究中的5例患者均采用VV-ECMO的转流模式，相较于VA-ECMO，VV-ECMO在术中可以提供全面的呼吸支持，清晰的手术视野以及更长的无通气时间，有利于外科医生的手术操作。然而，在ECMO使用过程中也存在一些并发症和局限性，最常见的是出血和血栓形成^[19,20]^，如弥散性血管内凝血、溶血、插管或手术部位出血、颅内出血以及胃肠道和肺部出血等。同时，ECMO使用过程中的缺氧和低灌注可能导致神经系统并发症，例如出血、癫痫发作和梗死。其他并发症还包括伴随感染、机械性损伤等^[21]^。在本研究中，仅有1例患者撤去ECMO后可能因全身肝素化引起凝血功能异常，术后第2天出现胸腔内滋养血管出血而二次手术止血。其余患者在撤去ECMO后均未出现出血、血栓形成及感染等ECMO相关并发症。

迄今为止，ECMO尚无标准化的抗凝管理策略。一项全球性研究^[22]^显示：96.7%的中心认为应全身抗凝，其中96.6%常规使用肝素抗凝；选择的监控标准主要为活化部分凝血活酶时间（41.8%）、ACT（30%）及抗Xa因子活性（22.7%）。差异的产生可能与地域差异有关，其中抗Xa因子活性监测更常应用于儿童。由于技术的进步，尤其是肝素涂层回路的发展，允许在某些情况下的ECMO支持使用低水平^[23]^或者无抗凝策略^[24]^。有的中心^[25]^报道了6例未进行全身抗凝的ECMO病例，部分患者在ECMO支持过程中应用肝素涂层管路而无肝素辅助，所有患者撤去ECMO后未出现出血等ECMO相关并发症及管道回路凝血。本研究中5例患者中2例采用了全身肝素化，另外3例未进行全身肝素化，仅通过ECMO肝素涂层管路维持。ECMO肝素涂层管路允许在一段时间内即使不额外使用抗凝药物也可以维持循环管路的正常功能，从而使具有高出血风险的患者获益^[26]^。因此，在选择抗凝策略时应根据患者的病情及客观情况来进行个性化设计。

我们的研究结果显示，VV-ECMO作为一种安全有效的氧合辅助手段，没有术后死亡发生，总体并发症发生率较低（20%）。仅有1例患者术后可能因凝血功能异常而出现胸腔内滋养血管出血，经手术止血后恢复出院。随访过程中有2例患者在术后第3年出现了肺转移瘤，经回顾文献，并无明确ECMO术后可引起远处转移的研究，因此我们认为转移不一定与ECMO使用存在相关性，需积累更多病例来进一步明确ECMO使用和气管肿瘤转移之间的关系。在这些病例中，我们积累的经验认为：（1）在ECMO的管路选择中，儿童插管应在超声下评估管径后视情况选择；成人常规选择动脉插管17#，静脉插管21#；当患者体重>90 kg时，一般动脉插管选择19#，静脉插管选择23#，当患者体重过大或氧合不足时，则也应通过超声来评估。我们认为置管部位一般选择右侧或左侧股静脉，右侧颈静脉；常规采用穿刺置管的方法。（2）在全身麻醉诱导成功后应立即进行ECMO导管置入和启动，在启动前需要充分扩容至少1000 mL。在VV-ECMO支持过程中，应避免使用交替通气，以确保手术进行顺畅，同时可以更快、更准确地完成缝合，从而维持患者的心肺稳定状态。术中中断通气后应避免再使用吸入麻醉药物，必须等待通气恢复后再继续使用。此外，ECMO可能会影响麻醉药物代谢，因此在手术过程中应持续监测麻醉深度并合理使用麻醉药。在ECMO支持期间，流量和氧合指标应维持在2.5-3.5 L/min和动脉血氧饱和度在85%-95%之间。当ECMO流量达到3.5 L/min仍无法满足氧合要求时，不应继续增加转流流量，因为继续加大氧合可能会引起红细胞破坏及凝血障碍，此时应考虑使用高频喷射通气来改善氧合。（3）在抗凝管理策略方面，我们中心选择了较低水平的抗凝方案（在ECMO插管前给予肝素50 IU/kg，根据ACT水平酌情考虑肝素化），若ECMO支持时间短于5 h，则无需特殊抗凝措施；若支持时间超过5 h，则应保持ACT在150-180 s。（4）撤机标准：在术中通气恢复后，应逐渐减少流量至1.0 L/min，随后监测动脉血气：若氧合指数>200，可考虑直接撤机；若氧合指数<200，则应在重症监护室继续ECMO支持治疗。在我们的研究中，大多数患者（80%, 4/5）在撤机后未发生感染、出血等并发症。

本研究存在一定的局限性。首先，本研究回顾性分析了ECMO在复杂TBS中的应用效果，但由于气管肿瘤及气管断裂的发病率较低，因此包含的病例数较少，虽然部分结果与国内外研究结论相近，但是研究结果存在较大的偏倚且普适性不强，循证医学价值较低，未来还需要包含更大样本量的研究。其次，由于本研究中病例不易获得，且部分患者处于紧急状态，ECMO在手术过程中不可或缺，因此无法为这类患者建立对照组。

综上所述，基于其有效的呼吸循环支持、良好的手术视野和麻醉管理等优势，VV-ECMO支持在复杂气道切除重建中具有显著的益处。

## References

[1] Liu J, Li S, Shen J, et al. Non-intubated resection and reconstruction of trachea for the treatment of a mass in the upper trachea. J Thorac Dis, 2016, 8(3): 594-599. doi: 10.21037/jtd.2016.01.56 27076957 PMC4805817

[2] Li S, Liu J, He J, et al. Video-assisted transthoracic surgery resection of a tracheal mass and reconstruction of trachea under non-intubated anesthesia with spontaneous breathing. J Thorac Dis, 2016, 8(3): 575-585. doi: 10.21037/jtd.2016.01.62 27076955 PMC4805803

[3] Ni Fhlatharta M, Khan A, Carton E, et al. Pre-emptive extracorporeal membrane oxygenation to support endobronchial stenting for severe airway obstruction. Eur J Cardiothorac Surg, 2021, 59(6): 1345-1346. doi: 10.1093/ejcts/ezaa425 33225355

[4] Pola Dos Reis F, Minamoto H, Bibas BJ, et al. Treatment of tracheal stenosis with extracorporeal membrane oxygenation support in infants and newborns. Artif Organs, 2021, 45(7): 748-753. doi: 10.1111/aor.13898 33350476

[5] Huang J, Huang W, Zhang J, et al. Application of laryngeal mask airway anesthesia with preserved spontaneous breathing in children undergoing video-assisted thoracic surgery. Front Pediatr, 2023, 11: 933158. doi: 10.3389/fped.2023.933158 36969299 PMC10036823

[6] He J, Xu X, Lan L, et al. End-to-side anastomosis in complex tracheal resection and reconstruction: a case series study. Transl Lung Cancer Res, 2022, 11(2): 165-172. doi: 10.21037/tlcr-22-32 35280311 PMC8902089

[7] Suzuki Y, Cass S, Lentz Carvalho J, et al. Extracorporeal membrane oxygenation for patients with thoracic neoplasms: an extracorporeal life support organization (ELSO) registry analysis. Ann Thorac Surg, 2022, 114(5): 1816-1822. doi: 10.1016/j.athoracsur.2022.03.030 35351418

[8] Macchiarini P. Primary tracheal tumours. Lancet Oncol, 2006, 7(1): 83-91. doi: 10.1016/s1470-2045(05)70541-6 16389188

[9] Zhao Y, Zhao H, Fan L, et al. Adenoid cystic carcinoma in the bronchus behaves more aggressively than its tracheal counterpart. Ann Thorac Surg, 2013, 96(6): 1998-2004. doi: 10.1016/j.athoracsur.2013.08.009 24094522

[10] Estephan J, Mercier O, Thomas de Montpreville V, et al. Retrospective study of outcomes after extended resection for tracheobronchial adenoid cystic carcinoma. J Thorac Cardiovasc Surg, 2023, 165(6): 1954-1964. e1955. doi: 10.1016/j.jtcvs.2022.10.048 36528436

[11] Ran J, Qu G, Chen X, et al. Clinical features, treatment and outcomes in patients with tracheal adenoid cystic carcinoma: a systematic literature review. Radiat Oncol, 2021, 16(1): 38. doi: 10.1186/s13014-021-01770-0 33608038 PMC7893857

[12] Etienne H, Fabre D, Gomez Caro A, et al. Tracheal replacement. Eur Respir J, 2018, 51(2): 1702211. doi: 10.1183/13993003.02211-2017 29444919

[13] Schmoekel NH, O’Connor JV, Scalea TM. Nonoperative damage control: the use of extracorporeal membrane oxygenation in traumatic bronchial avulsion as a bridge to definitive operation. Ann Thorac Surg, 2016, 101(6): 2384-2386. doi: 10.1016/j.athoracsur.2015.08.033 27211954

[14] Clark J, Morrison JJ, O’Connor JV. Extracorporeal membrane oxygenation support during repair of a noniatrogenic tracheal injury. Ann Thorac Surg, 2022, 113(1): e49-e51. doi: 10.1016/j.athoracsur.2021.03.029 33774000

[15] Slama A, Stork T, Collaud S, et al. Current use of extracorporeal life support in airway surgery: a narrative review. J Thorac Dis, 2023, 15(7): 4101-4110. doi: 10.21037/jtd-22-1483 37559597 PMC10407487

[16] Hoetzenecker K, Klepetko W, Keshavjee S, et al. Extracorporeal support in airway surgery. J Thorac Dis, 2017, 9(7): 2108-2117. doi: 10.21037/jtd.2017.06.17 28840012 PMC5542968

[17] Rinieri P, Peillon C, Bessou JP, et al. National review of use of extracorporeal membrane oxygenation as respiratory support in thoracic surgery excluding lung transplantation. Eur J Cardiothorac Surg, 2015, 47(1): 87-94. doi: 10.1093/ejcts/ezu127 24659317

[18] Spaggiari L, Galetta D, Lo Iacono G, et al. Robotic-assisted tracheal resection for adenoid cystic carcinoma with extracorporeal membrane oxygenation support. JTCVS Tech, 2023, 21: 244-246. doi: 10.1016/j.xjtc.2023.07.016 37854844 PMC10580088

[19] Rob D, Špunda R, Lindner J, et al. A rationale for early extracorporeal membrane oxygenation in patients with postinfarction ventricular septal rupture complicated by cardiogenic shock. Eur J Heart Fail, 2017, 19 Suppl 2: 97-103. doi: 10.1002/ejhf.852 28470920

[20] Nunez JI, Gosling AF, O’Gara B, et al. Bleeding and thrombotic events in adults supported with venovenous extracorporeal membrane oxygenation: an ELSO registry analysis. Intensive Care Med, 2022, 48(2): 213-224. doi: 10.1007/s00134-021-06593-x 34921625 PMC9178906

[21] Teijeiro-Paradis R, Gannon WD, Fan E. Complications associated with venovenous extracorporeal membrane oxygenation - what can go wrong?. Crit Care Med, 2022, 50(12): 1809-1818. doi: 10.1097/ccm.0000000000005673 36094523

[22] Protti A, Iapichino GE, Di Nardo M, et al. Anticoagulation management and antithrombin supplementation practice during veno-venous extracorporeal membrane oxygenation: a worldwide survey. Anesthesiology, 2020, 132(3): 562-570. doi: 10.1097/aln.0000000000003044 31764152

[23] Koryllos A, Lopez-Pastorini A, Galetin T, et al. Use of extracorporeal membrane oxygenation for major cardiopulmonary resections. Thorac Cardiovasc Surg, 2021, 69(3): 231-239. doi: 10.1055/s-0040-1708486 32268398

[24] Takagaki M, Yamaguchi H, Ikeda N, et al. Post-cardiotomy venovenous extracorporeal membrane oxygenation without heparinization. Gen Thorac Cardiovasc Surg, 2019, 67(11): 982-986. doi: 10.1007/s11748-018-0990-2 30120673

[25] Fina D, Matteucci M, Jiritano F, et al. Extracorporeal membrane oxygenation without systemic anticoagulation: a case-series in challenging conditions. J Thorac Dis, 2020, 12(5): 2113-2119. doi: 10.21037/jtd.2020.04.54 32642115 PMC7330289

[26] Van Sant L, Giuliani S, Mitchell J. Evolving role for extracorporeal membrane oxygenation (ECMO) in trauma patients. Int Anesthesiol Clin, 2021, 59(2): 31-39. doi: 10.1097/aia.0000000000000313 33710001

